# Rivaroxaban does not impair fracture healing in a rat femur fracture model: an experimental study

**DOI:** 10.1186/s12891-015-0502-9

**Published:** 2015-04-09

**Authors:** Tim Klüter, Matthias Weuster, Stefan Brüggemann, Leif Menzdorf, Stefanie Fitschen- Oestern, Nadine Steubesand, Yahya Acil, Thomas Pufe, Deike Varoga, Andreas Seekamp, Sebastian Lippross

**Affiliations:** Department of Trauma Surgery, University Medical Center of Schleswig-Holstein, Campus Kiel, Germany; Department of Anatomy and Cell Biology, RWTH Aachen University, Aachen, Germany; Department of Maxillo-Facialy, University Medical Center of Schleswig-Holstein, Campus Kiel, Germany; Department of Trauma Surgery, University of Kiel, Arnold-Heller-Strasse 3, 24105 Kiel, Germany

## Abstract

**Background:**

The prescription of the oral anticoagulant rivaroxaban to prevent thromboembolic episodes associated with orthopaedic surgery has dramatically increased since it was introduced. Rivaroxaban is beeing prescribed although recent in-vitro studies revealed that it impaired osteoblast metabolism. In this study we analysed the effect of rivaroxaban on fracture healing in a rat femur fracture model.

**Methods:**

Femur fractures were created by a 3-point-bending device in 48 Wistar rats and subsequently stabilized by intramedullary nailing. After the surgical procedure animals were randomised into four groups. Two groups were fed with 3 mg rivaroxaban per kg body weight per day and two control groups were fed with chow only. Animals were euthanized 28 or 49 days after surgical procedure. Femurs underwent undecalcified histologic staining micro CT scanning and biomechanical testing. The statistical significance was evaluated using one-way Anova with Bonferroni correction.

**Results:**

Micro CT-scans revealed significantly increased volume of bone tissue in the fracture zone between day 28 and 49. During the same time callus volume decreased significantly. Comparing the fracture zone of the rivaroxaban group to the control group the treated group revealed a larger callus and a marginal increase of the tissue mineral density. The torsional rigidity was not influenced by the treatment of rivaroxaban.

**Conclusion:**

In the present study we were able to demonstrate that rivaroxaban does not impair fracture healing in a rat femur fracture model. Considering the fact that low molecular weight heparins delay fracture healing significantly, rivaroxaban might be an improved alternative.

## Background

Thrombembolic complications constitute a main cause of mortality after fractures of the lower limb. Several studies document that without thromboprophylaxis the incidence of venous thromboembolism (VTE) after hip fracture, total hip arthoplasty or multiple trauma goes up to one third to one half [1,2]. Thromboprophylaxis using heparin can reduce thrombembolic complications by up to 40 to 60% [3]. During the last decades low molecular weight (LMW) heparins replaced unfractionated heparin (UH) and became gold standard for thromboprophylaxis in orthopaedic surgery [4].

In 1956 Stinchfield and colleagues documented the effects of heparin and warfarin on bone repair. They described that daily administration of heparin causes attenuated fracture healing in rabbit and canine models [5]. Street et al. analyzed the effect of LMW heparin on fracture healing of the rabbit. Histomorphometric, histologic and immunohistochemical testings demonstrated that the bone repair was attenuated at all times in animals receiving Enoxaparin [6]. Besides LMW heparins have been shown to cause osteoporosis when given for longer than 8 weeks [7]. In case of fractures, pericytes migrate from peristostal vasculation and transform into ostogenic progenitor cells. UH and LMW heparin bind to endothelial cells and osteoblast, reduce their activity and prohibit neovascularisation [8]. On the other hand decreased blood clotting due to an enlarged fracture hematoma has a negative influence on the initial phase of the fracture healing. Potassium channels in osteoblasts and endothelial cells are influenced negatively by hyperkalaemia, by enabling them to react on proliferative cytokines. This leads to significant cytostasis and cell distraction in the fresh hematoma [6,9]. Both mechanisms, the binding of endothelial cells and osteoblasts as well as the bleeding tendency may cause a delay of fracture healing.

Recently, the novel oral anticoagulant rivaroxaban (Xarelto^©^) was developed to safely and effectively prevent VTE. Rivaroxaban inhibits coagulation factor Xa directly by binding to its active centre. Factor Xa is the active form of the coagulation factor thrombokinase and is synthesised in liver. Long et al. have recently shown that in comparison with LMW heparins rivaroxaban reduced the incidence of VTE by 45% without increasing the risk of bleeding in patients with lower extremity fractures [10]. Patients receiving rivaroxaban do not need to undergo daily injection or regular drug monitoring and therefore profit from a higher comfort and greater compliance. During the last years oral factor Xa inhibitors gained continuously more popularity in thromboprophylaxis after total hip and knee arthroplasty. It is assumed that oral factor Xa inhibitors will be established in thromboprophylaxis of trauma patients soon.

So far the effect of rivaroxaban on fracture healing has not been investigated. In in vitro studies Gigi et al. have recently revealed an inhibition of osteoblast metabolism caused by rivaroxaban [11]. Considering these facts the authors hypothesized that thromboprophylaxis with this new substance would promote interfragmentary hematoma collection and delay bone formation during fracture healing. Therefore we investigated the effect of rivaroxaban in an established rodent fracture model, first published by Bonnarens [12]. The application and dose is well investigated because first developments and tests were conducted in rats [13].

## Methods

### Animals

Ethical approval was obtained from the regional Ethics committee Schleswig-Holstein, Germany (V 312-72241.121-9 (8-1/09)). 48 female Wistar-rats weighing 230 ± 30 g were obtained from the local Service Unit of the authors’ institution. The animals were kept two per cage with free access to rat-chow and water. The lighting was maintained on a 12-hour light-dark cycle. Analgesic treatment was administered to all animals 4 times a day via drinking water (100 mg/kg metamizole sodium (Novalgin®, Hoechst, Unterschleißheim, Germany)). After the surgical procedure four groups were randomised, two groups were treated with 3 mg rivaroxaban per kg body weight per day together with their chow, two control groups were only fed with chow (Table 1). The animals were euthanized on day 28 or 49 after surgical procedure.

**Table 1:** **Group distribution of 48 rats**

**Time point**	**Test**	**Quantity**
**Day 28**	Biomechanical testing	6 rats each group
	Micro CT analysis	6 rats each group
	Histologic assessment	
**Day 49**	Biomechanical testing	6 rats each group
	Micro CT analysis	6 rats each group
	Histologic assessment	

### Surgical procedure

Implants and surgical equipment were sterilized in an autoclave. Sterile gowns, gloves, surgical mask and theatre caps were used. Preoperatively each rat received 0.06 mg buprenorphine orally. The animals were anaesthetized with Fentanyl 0.005 mg/kg, Midazolam 2.0 mg/kg and Medetomedine 0.15 mg/kg by intraperitoneal injections. The rat leg was shaved and washed with chlor-hexidine. Medial to the patella the skin was incised and the patella dislocated to the lateral side, so that the condyles were exposed. A 0.90x40 mm sterile cannula (Braun, Melsungen, Germany) was drilled though the medullary channel using an intercondylar approach. The sharp end was place to the proximal end of the femur and the rest of the cannula was cut off, so that no end extended beyond the bone. The patella was repositioned over the knee joint and the skin was closed with resorbable thread. A closed middiaphyseal transverse fracture was created in the right femur by three-point bending as described by Bonnarens [12]. Anaesthesia was antagonized by intraperitoneal Naloxon 0.12 mg/kg, Flumazenil 0.2 mg/kg und Atipamezol 0.75 mg/kg. Animals were sacrificed by CO_2_ asphyxation on day 28 and day 49. Bones were dissected, fixed in 4% paraformaldehyde and stored in 70% Ethanol or used for biomechanical testing.

### Biomechanical testing

Torsional rigidity was analysed as described by Manigrasso [14]. Testing was performed using a custom built torsional servohydraulic testing device as described by Turner et al. and Engesaeter et al. [15,16] using a 20 Nm reaction torque load cell. Femurs were tested to failure at an actuator head displacement rate of 1°/second. The peak torque and angle at failure were measured from the torque-angle deflection curves.

### Micro CT analysis

For a detailed qualitative and quantitative 3-D evaluation, the fracture site was examined in a micro CT scanner (Scanco Medical AG, Bassersdorf, Switzerland) equipped with a 70 μm focal spot microfocus X-ray tube as a source. During scanning, the femoral bones were placed in phosphate buffered saline. Radiographic projections were performed at 70 kV and 114 μA with a fixed integration time of 200 ms. For image acquisition, the specimen was mounted on a turntable shifted automatically in an axial direction over 180°, taking 500 projections. Three-dimensional images were stored in 3-D arrays with an isotropic voxel size of 16 μm and a pixel size of 1024x1024. The region of interest (ROI) covered a total range of 8.4 mm (encompassing 4.2 mm proximal and distal to the fracture line). For quantitative analysis of bone formation within the ROI Scano Evaluation software (Scano Medical, Brüttisellen, Switzerland) was used to obtain the volume of interest. Depending on the grey threshold these volumes were reflected to highly mineralised tissue (bone, 33 to 60%) and low mineralised tissue (callus, 17.5 to 33%). Tissue mineralized density was calculated by dividing the volume of the mineralised callus by the tissue’s density. Due to technical problems the results of 3 rats treated for 49 days were deleted somehow. When this error was noticed, samples could not be scanned again.

### Preparation of specimens

To illustrate the micro-architecture of the callus, undecalcified bone histology was performed. The bones were fixed in 10% neutral buffered formalin for 14 days at room temperature, dehydrated with ascending concentration of ethanol and embedded in methylmetacrylate for 5 days at 4°C. Afterwards tissue was soaked in methylmethacrylat monomer, nonylphenyl-polyethyleneglycol acetate and azoisobutyronitrile (all Sigma-Aldrich, St. Louis, USA). The blocks were released from the glass vials and undecalcified sections of 40 μm were sawed and grinded using an EXAKT diamond saw system (Exakt, Norderstedt, Germany).

### Histologic assessment

For histologic staining sections were lubricated with 0.1% formic acid for 2 minutes and washed with aqua afterwards. The samples were immersed in 20% methanol for 1 hour and stained with toluidine blue or hematoxylin and eosin.

### Statistical analysis

Statistical analysis was performed using a standard software application (SPSS Inc., Chicago, IL, USA). Results of CT scanning and torsional testing were expressed as the means ± standard deviation of tested groups. The different groups were tested for normality using Kolmogorov-Smirnov test. Statistical significance was evaluated using one-way Anova with Bonferroni correction. Sample distribution for torsional testing and CT scanning were illustrated with Box and whisker plots.

## Results

All animals recovered quickly after the surgical procedures and fully mobilized within two days. No signs of pain or dysfunction in the physical motion was recognized. No animal had to be excluded because of death, implant dislocation or soft tissue infection.

### Histology

**Figure 1:**
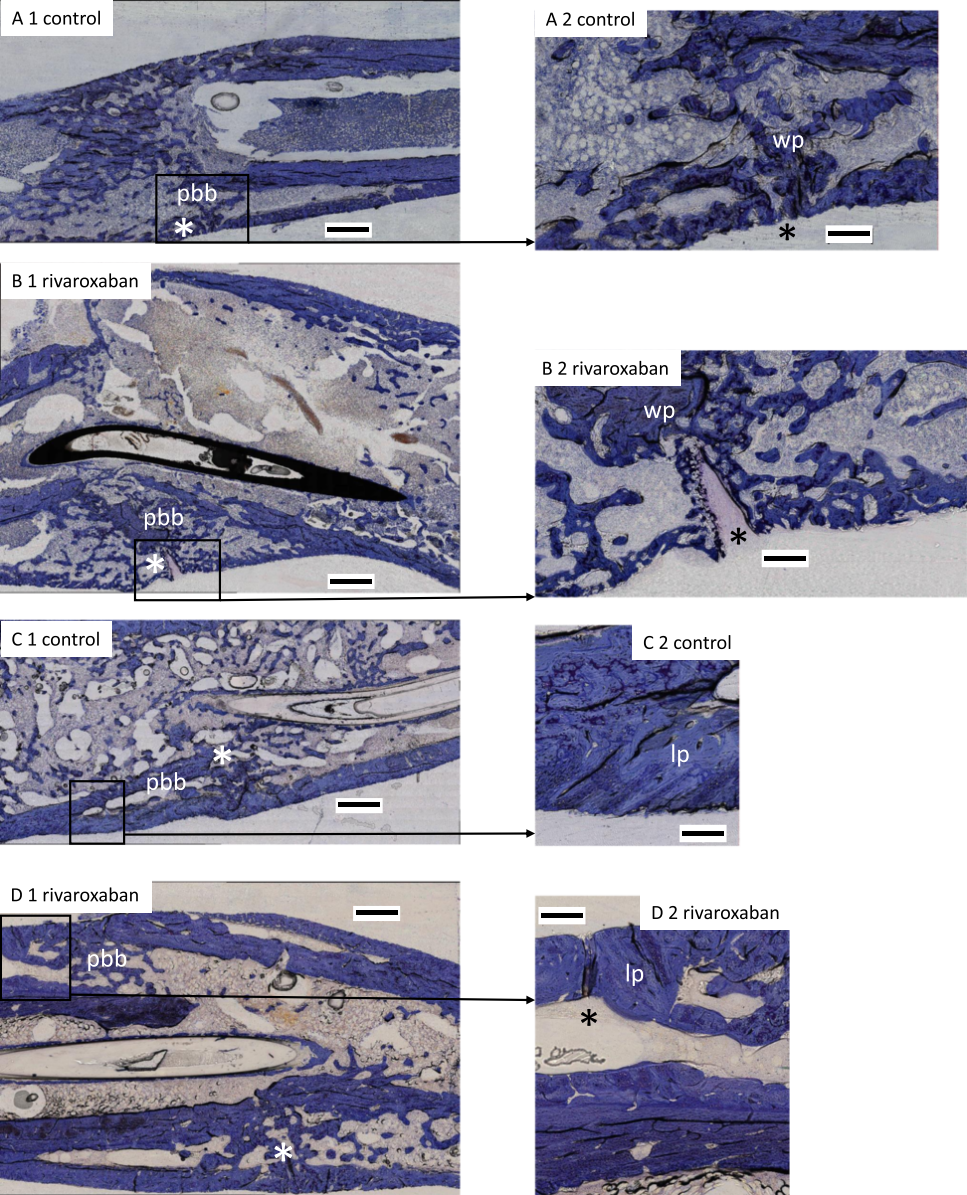
**Photomicrography of fracture callus from rat femur.** Panoramic views (1) and high magnification (20×) photomicrography (2) of fracture healing and callus formation was illustrated using calcified bone histology technique with toluidine blue staining. The magnification points up the remodelling zone within the fracture gap. 28 days after fracture the fracture gap (*) and a sufficient callus is noticeable with partial bony bridging (A + B). 49 days after fracture the femurs demonstrate increased bone tissue with lamellar bone (C + D). No significant difference could be seen between control and treatment group. Bar represents 1 mm **(A1 – D1)** or 100 μm **(A2 – D2)** respectively. pbb = partial bony bridging; lp = lamellar part; wp = woven part.

For assessment of fracture healing and callus formation we used a standard calcified bone histology technique. Thereby we could illustrate the cellular and mineralised components of the callus in the longitudinal axis, which provides vital information on bone turnover or bone formation and resorption. 28 days after closed femur fracture the fracture gap was still visible and sufficient callus had arisen (Figure 1). Tolouidine blue staining showed partial bony bridging of the gap in the 28 days group and the bridging increased 49 days after fracture. During the remodelling processes of the endosteal bony callus the amounts of newly formed bone increased between day 28 and 49. Bony callus consists of woven and lamellar bone, and here the lamellar part increased and the woven part decreased.

No significant difference in fracture healing could be identified in either comparison group after 28 or 49 days. Over all the callus sizes showed great variability amongst all groups.

### Biomechanical analysis

**Figure 2:**
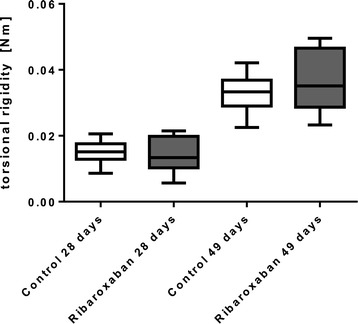
**Torsional rigidity.** Torsional rigidity of 28-days- and 49-day-old fractures callus species was detected for control and rivaroxaban treated rats. Rigidity raised between day 28 and 49, but no significant difference was measured between control and treatment group. n = 6 (each group).

The operated femurs were analysed for torsional rigidity 28 and 49 days after the surgical procedure. Parameters such as torsional rigidity calculated from torsion tests are convenient for determining the structural and material properties of the healing tissue. After 4 weeks of rivaroxaban diet the femur callus showed no significant difference in torsional rigidity compared to the control group (0.0154 ± 0.0039 N/m^2^ versus 0.0141 ± 0.0056 N/m^2^; p = 0.81). Even after 7 weeks of rivaroxaban treatment rigidity did not change significantly (Figure 2).

### Micro CT scan of interfragmentary zone

**Figure 3:**
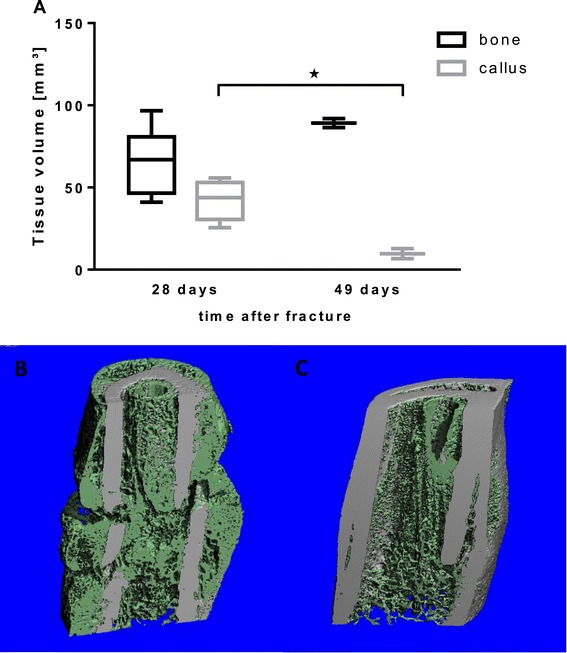
**3D imaging of the interfragmentary zone. (A)** Tissue volumes of defined fracture zone of rats’ femur treated with rivaroxaban was determined by micro-CT analysis. During Fracture healing volume of bone tissue increased from 65.9 mm^3^ to 89.2 mm^3^. On the other hand callus volume decreased from 42.17 mm^3^ to 9.7 mm^3^. B + C The images show femurs of rivaroxaban treated rats 28 and 49 days after femur fracture. **(B)** 28-days-old femurs fracture reveals a fracture gap a sufficient callus (green). **(C)** 49 days after fracture the volume of callus tissue (green) had decreased and formatted to mineralised bone tissue.

**Figure 4:**
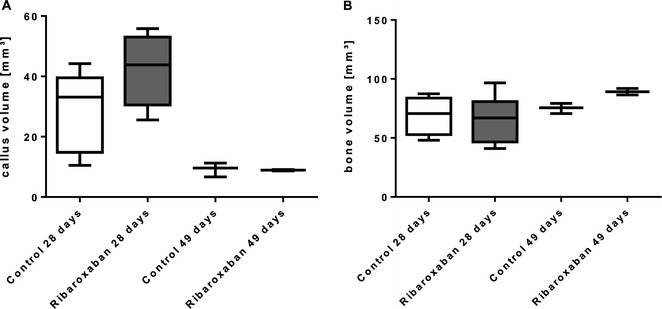
**Tissue volumes. (A)** Femur fracture callus volume of control and rivaroxaban treated rats after 28 and 49 days was detected by micro CT-scan. Treated rats showed increase of callus volume compared to control group. 49 days after fracture callus was almost transformed to bone tissue. **(B)** Bone volume decreased from day 28 to day 49, but no significant differences could be seen between the groups. n = 6 (28 days); n = 3 (49 days).

The callus volume was determined from 4.2 mm proximal to 4.2 mm distal of the fracture gap by micro CT scanning. During fracture healing bone tissue increaed in rivaroxaban treated rats from 65.9 ± 8.1 mm^3^ to 89.2 ± 2.7 mm^3^ (p = 0.06), whereas callus volume decreased from 42.17 ± 5.3 mm^3^ to 9.7 ± 3.1 mm^3^ (p = 0.04; Figure 3). After 28 days we detected an insignificant increase of callus volume of specimens which underwent rivaroxaban treatment compared to the control animals (28.37 ± 13.42 mm^3^ versus 42.17 ± 11.98 mm^3^; p = 0.12). After 49 days the callus was almost completely transformed to bony tissue, thus no significant alteration between both groups could be determined (Figure 4A).

No significant difference could be seen in bone volume between the groups at both points of time (69.0 mm^3^ ± 17.0 versus 65.9 mm^3^ ± 19.8 and 75.2 mm^3^ ± 4.3 versus 89.15 mm^3^ ± 3.9) (Figure 4B).

### Tissue mineral density (TMD)

**Figure 5:**
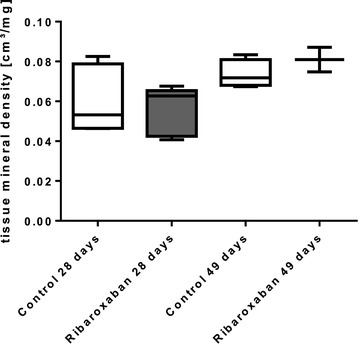
**Tissue mineral density.** Tissue mineral density of rat femur callus was quantified by micro CT-scan 28 and 49 days after femur fracture. The rivaroxaban treatment group revealed marginal increase compared to control group. n = 6 (28 days); n = 3 (49 days).

To quantify the formation of the callus we analysed the TMD which was generated by micro CT scans within the defined area. After 28 days of rivaroxaban treatment we documented a marginal increase of the TMD compared to the control specimens (0.057 ± 0.017 mg/cm^3^ versus 0.055 ± 0.012 mm^3^; p = 0.31). A similar trend was ascertained for 49-day-old callus (0.08 ± 0.008 mg/cm^3^ versus 0.076 ± 0.007 mg/cm^3^; p =0.86), but both effects were statistically insignificant (Figure 5).

## Discussion

Previous studies indicate a delay of callus remodelling due to thromboprophylaxis with UH and LMW heparin [9,17]. So far there is no study which investigated the effect of the new oral Factor Xa inhibitor rivaroxaban on fracture healing. Considering the fact that this anticoagulant is finding its way into treatment of trauma patients, it is indispensable to know its influence on the healing bone. Due to the complexity of interaction of the diverse cell types and tissues in vitro reproducibility of the bone healing process is not convincing. Therefore we utilised a classic established rodent fracture model [12].

In contrast to previous studies with heparins no delay of fracture healing relating to rivaroxaban treatment could be determined in the present study. Daily administration of the oral factor Xa inhibitor increased the callus volume insignificantly. To evaluate the progress of bone healing, we analysed callus by histological staining and documented a comparable repair process concerning the patterns of bone bridging, including periosteal, endosteal, intercortical patterns and mineralisation [18] We did not identify significant differences within the two groups at the same points of time. To quantify the callus mineralisation we performed micro CT scans as a valid assessment for bone stiffness. Previous studies revealed that TMD has the same validity for stiffness as the torsional righty [19-21]. The high resolution scan of the callus microstructure showed a small and not significant increase of TMD after rivaroxaban treatment. These results correlate with an in vitro study with cultured osteoblast which demonstrated a dose-dependent transient inhibition of osteoblast metabolism by rivaroxaban but no effect on mineralisation [11]. As another parameter for bone stiffness we measured the bone volume which did not increase significantly.

The torsional rigidity is a scientifically recognized standard to evaluate fracture healing [6,20,22]. Our study indicates that the torsional rigidity did not decrease after application of the oral anticoagulant. In contrast to that in previous studies LWM heparins result in a decrease of torsional stiffness and lower energy to fracture [6]. The authors explained this effect by binding of enoxaparin to vascular endothelium which disrupts callus vascular assembly and also by an increase of interfragmentary hematoma and subsequent cytotoxic effects on cells of the medullary callus. Due to technical problems we could not reach the minimal quantity to perform statistical analysis for the 49-days-old treatment group. Eventually the existing results could not justify further animal experimentation to reach statistical significance.

Taken together we could not document a negative effect in bone stiffness after rivaroxaban treatment. The higher callus volume on day 28 in combination with equal bone volume and tissue mineral density seems not to have an influence on the stability of the fracture.

## Conclusion

In the present study we could for the first time demonstrate that thromboprophylaxis with rivaroxaban does not downgrade fracture healing in a rodent fracture model. Because of the low number of random samples, we are aware that this study is not powerful enough to give a general recommendation for the use of oral factor Xa inhibitor in case of trauma patients. Nevertheless considering the fact that LMW heparin, the gold standard for thromboprophylaxis, delays fracture healing significantly, rivaroxaban might be an improved alternative. The indications in prevention and treatment of thrombosis, particularly in trauma surgery, need further evaluation covering all aspects of bone repair.
